# Supplementary figures and images for: Plasmodium falciparum malaria drives epigenetic reprogramming of human monocytes toward a regulatory phenotype
Source: PLoS Pathog. 2021 Apr 6;17(4):e1009430. doi: 10.1371/journal.ppat.1009430 (PMC8023468; doi:10.1371/journal.ppat.1009430)

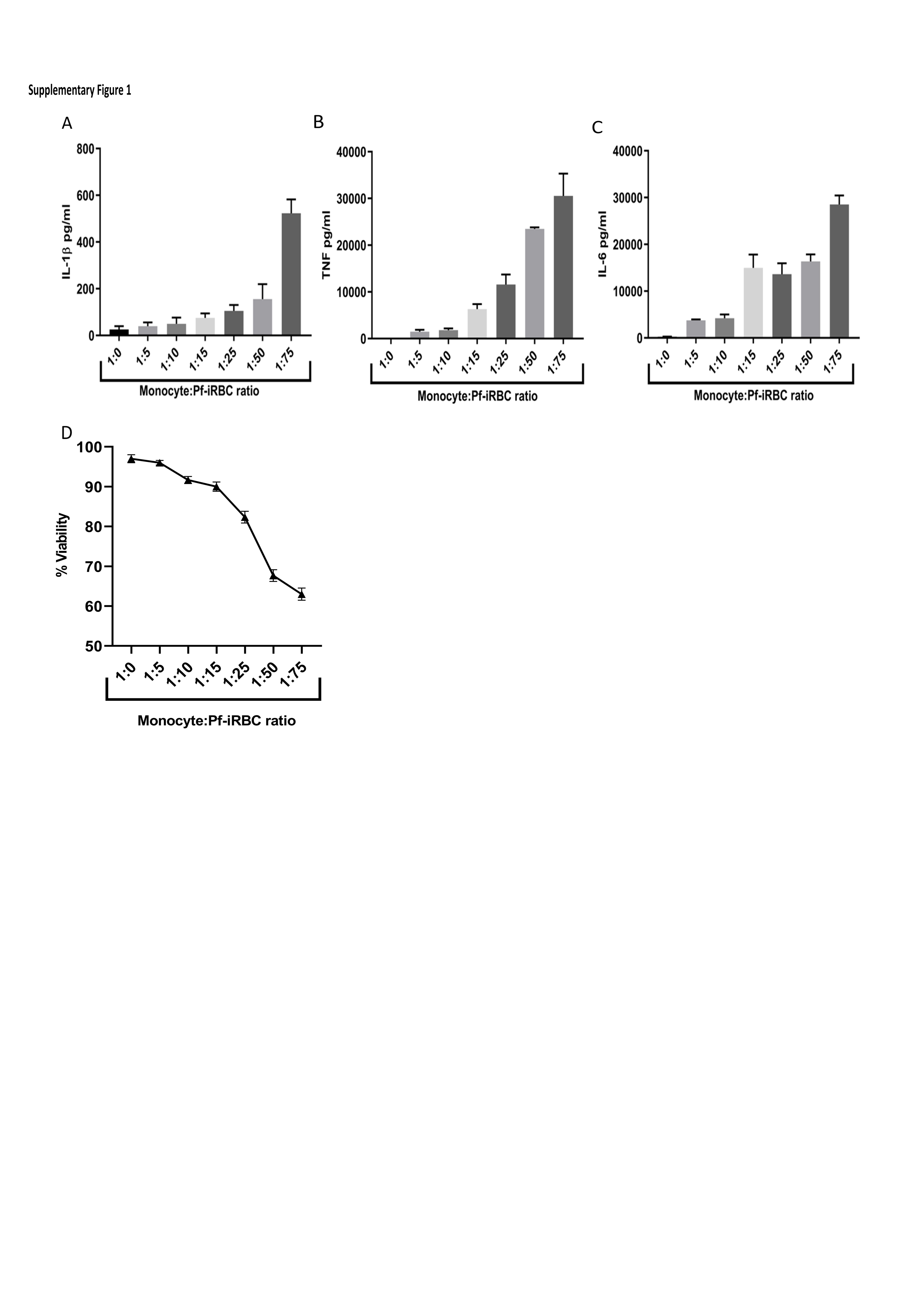

Supplement: S1 Fig — Elutriated monocytes from healthy U.S. adults (n = 3) were co-cultured with increasing concentrations of Pf-iRBCs. After 24 hours, IL-1β (A), TNF (B) and IL-6 (C) were measured in supernatants, and (D) cell viability was determined by trypan blue dye exclusion and expressed as percent viability. (TIF) [file ppat.1009430.s001.tif]

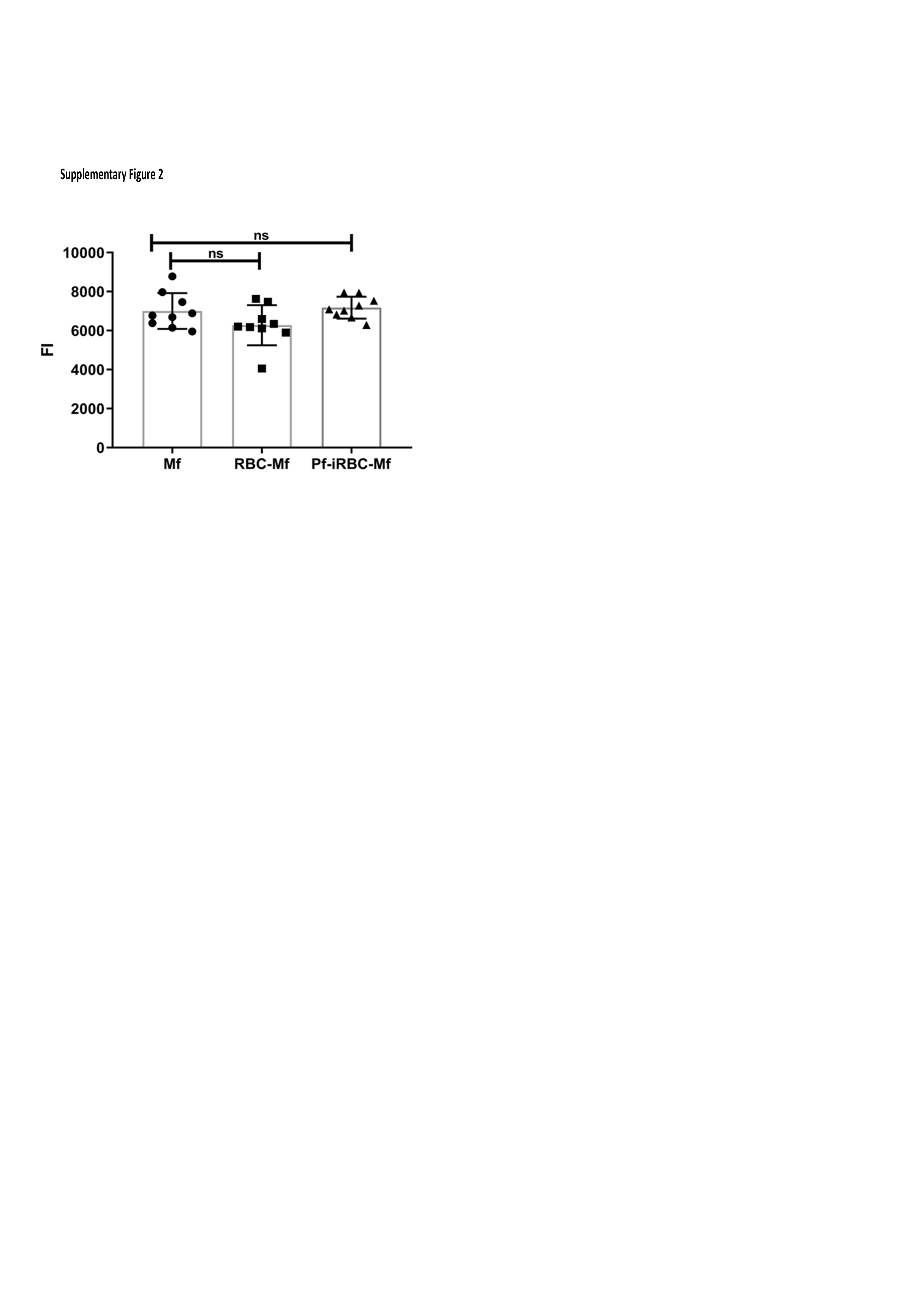

Supplement: S2 Fig — Elutriated monocytes from healthy U.S. adults (n = 9) were incubated for 24 hours with medium alone, uninfected red blood cells (RBC) or Pf-iRBC (monocyte:Pf-iRBC ratio 1:15). At 24 hours, cells were washed and incubated for 3 additional days in human serum plus medium to allow monocytes to differentiate into macrophages (Mf). To quantify cell viability on day five, 10% v/v alamarBlue HS was added to the culture medium of the three populations of macrophages (Mf, RBC-Mf and Pf-iRBC-Mf) for 5 hours and fluorescence intensity (FI) was measured according to the manufacturer’s instructions. FI was normalized to the fluorescence signal in media without cells. Data were analyzed by the Wilcoxon test with Bonferroni adjustment, and levels of significance between the groups are indicated. (TIF) [file ppat.1009430.s002.tif]

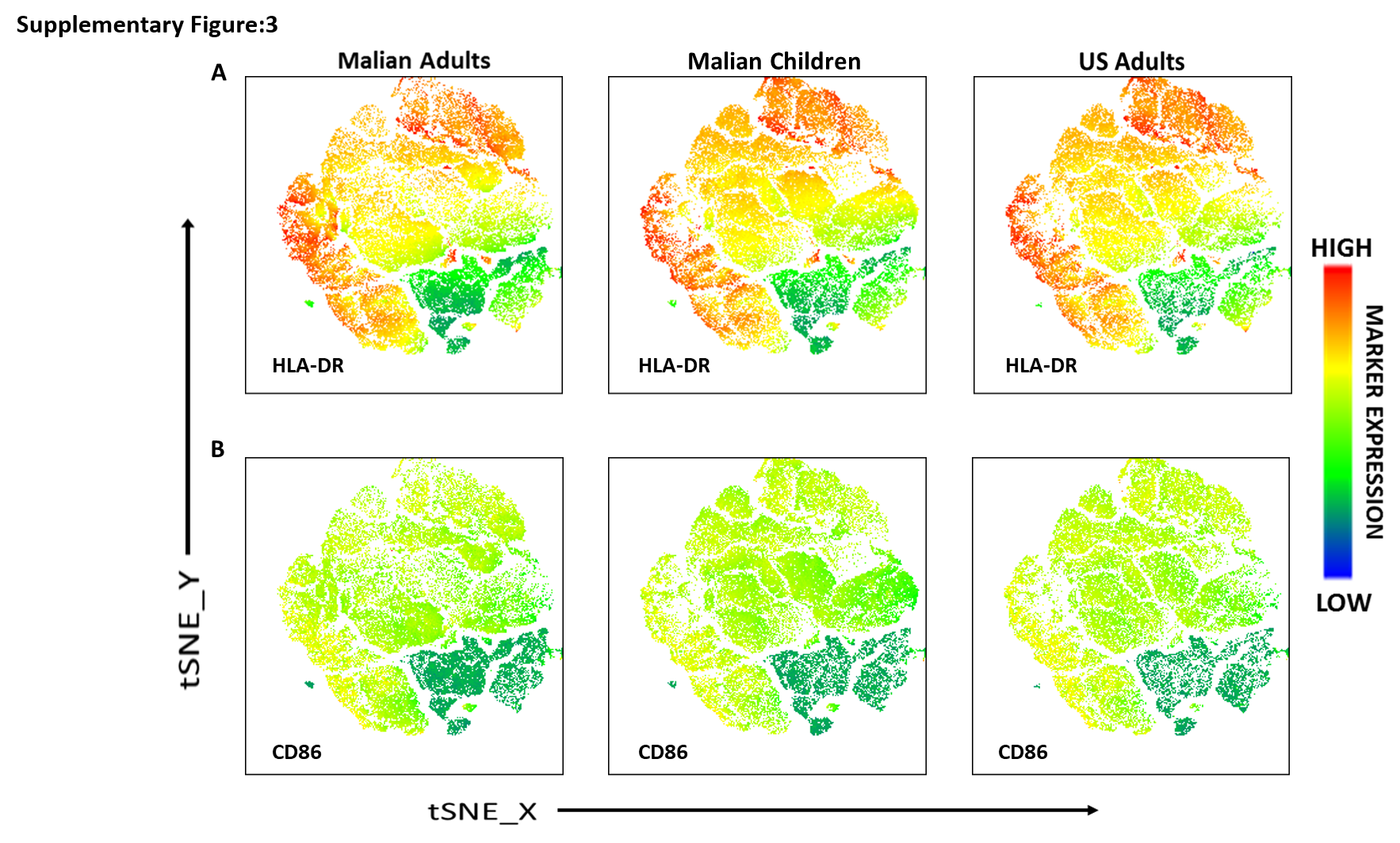

Supplement: S3 Fig — PBMCs from Malian children (aged 4–6 years; n = 9) and adults (n = 9) before the malaria season, as well as healthy malaria-naïve U.S. adults (n = 7) were gated for live monocytes and analyzed ex vivo by flow cytometry for surface expression of (A) HLA-DR and (B) CD86. t-SNE analysis of monocytes for all subjects in each group. Expression of each marker is indicated by a color scale. (TIF) [file ppat.1009430.s003.tif]
